# Are *Juglans neotropica* Plantations Useful as a Refuge of Bryophytes Diversity in Tropical Areas?

**DOI:** 10.3390/life11050434

**Published:** 2021-05-12

**Authors:** Jefferson Medina, Wilson Quizhpe, Jorge Déleg, Karina Gonzalez, Zhofre Aguirre, Nikolay Aguirre, Luis Montaño, Ángel Benítez

**Affiliations:** 1 Maestría en Biología de la Conservación y Ecología Tropical, Universidad Técnica Particular de Loja, San Cayetano s/n, Loja 1101608, Ecuador; jeffersonmedinabenitez@gmail.com (J.M.); jorgo31@hotmail.com (J.D.); 2 Docente Investigador, Facultad de Ciencias de la Vida, Universidad Estatal Amazónica Sede El Pangui, El Pangui 190401, Ecuador; wr.quizhpec@uea.edu.ec; 3 Carrera de Ingeniería Forestal, Universidad Nacional de Loja, Loja 110111, Ecuador; karogonzalezvaldiviezo@gmail.com; 4 Docente Investigador, Universidad Nacional de Loja, Loja 110111, Ecuador; zhofre.aguirre@unl.edu.ec (Z.A.); nikolay.aguirre@gmail.com (N.A.); 5 Titulación en Gestión Ambiental, Universidad Técnica Particular de Loja, San Cayetano s/n, Loja 1101608, Ecuador; luis46vr58@gmail.com; 6 Biodiversidad de Ecosistemas Tropicales-BIETROP, Herbario HUTPL, Departamento de Ciencias Biológicas, Universidad Técnica Particular de Loja, San Cayetano s/n, Loja 1101608, Ecuador

**Keywords:** montane forest, epiphyte, canopy openness, richness, deforestation

## Abstract

Neotropical montane forests are considered biodiversity hotspots, where epiphytic bryophytes are an important component of the diversity, biomass and functioning of these ecosystems. We evaluated the richness and composition of bryophytes in secondary successional forests and mixed plantations of *Juglans neotropica*. In each forest type, the presence and cover of epiphytic bryophytes was registered in 400 quadrats of 20 cm × 30 cm. We analyzed the effects of canopy openness, diameter at breast height (DBH) and forest type on bryophyte richness, using a generalized linear model (GLM), as well as the changes in species composition using multivariate analysis. Fifty-five bryophyte species were recorded, of which 42 species were in secondary forests and 40 were in mixed plantations. Bryophyte richness did not change at forest level; however, at tree level, richness was higher in the mixed plantation of *J. neotropica* compared to the secondary forests, due to the presence of species adapted to high light conditions. On the other hand, bryophyte communities were negatively affected by the more open canopy in the mixed plantation of *J. neotropica*, species adapted to more humid conditions being less abundant. We conclude that species with narrow microclimatic niches are threatened by deforestation, and *J. neotropica* plantations do not act as refuge for drought-sensitive forest species present in secondary forests.

## 1. Introduction

Neotropical forests are the most diverse in the world, occupying an area of about 48 million hectares, approximately 50% of which are located in South America [1]. A large part of this diversity is located in the Andean montane or cloud forests [2,3]. Ecuador is country with the second largest area of this type of forest in South America (11,200,000 ha), of which only 1,448,700 hectares are protected [4], despite being considered biodiversity hotspots [5,6]. These forests support a wide range of biological resources and provide ecosystem services, mainly related to water, climate regulation and carbon capture and storage [7,8].

Ecuador has the highest deforestation rates in South America, estimated at 1.8% during the 2001–2010 period [9]. By 2008, around 46% of southern Ecuador’s original forest cover had been converted into other land use types (e.g., pastures) [10], generating important economic and social consequences [11]. Loss of forest cover, alteration of the hydric balance [12,13], and habitat fragmentation [14] are the main drivers of changes in the composition, structure and functionality of these ecosystems [15,16], determining biodiversity loss [17]. On the other hand, forest plantations occupy about 187 million hectares worldwide, with an annual increase of 4.5 million hectares [18], with South America leading in terms of new planted surface. In Ecuador, especially in the mountainous region (Sierra), pine trees and eucalypts have been widely used for forest plantations [19,20]. As a result, areas of primary and secondary forests are surrounded by these anthropized ecosystems, including a few areas with *Juglans neotropica* plantations.

One of the ecological groups most affected by deforestation are epiphytes (both vascular and non-vascular), because they are very sensitive to environmental changes [21,22]. Epiphytes constitute an essential part of montane forests in terms of diversity and functionality [21,23,24]. An important element of this group are the bryophytes, which play a key role in the functioning and balance of ecosystems [25]; because of their physiological and morphological characteristics (poikilohydric organisms), they are narrowly adapted to humidity, solar radiation and temperature conditions, which is why they have become a model group to evaluate forest disturbance [22,26,27].

Forest plantations are anthropized ecosystems widely distributed in the tropical Andes, which is why they have been the subject of different research projects related to flora diversity. Several studies show that diversity decreases drastically in plantations when compared to natural forests [28,29]; however, there are also studies indicating that there are no differences in diversity between primary forests and plantations [30,31,32,33], suggesting that organisms respond differently in these anthropized systems [34]. In some tropical areas, the relationship of bryophyte diversity in agroforestry systems has been documented in plantations of *Theobroma cacao* [35,36,37,38] and of the genera *Citrus* and *Mangifera* [39]. In addition, research has been carried out in monospecific forests of the genera *Polylepis* [40,41], *Quercus* [25,42], *Nothofagus* [43,44] and *Alnus* [22], but the role of *J. neotropica* plantations as reservoirs of bryophyte biodiversity has not been documented to date. Thus, the present research is aimed at comparing for the first time the diversity of epiphytic bryophytes in mixed plantations of *J. neotropica* to secondary montane forests. The hypothesis is based on the observation that more canopy openness in plantations could determine changes in the richness and composition of bryophytes, as has been shown in previous research, comparing agroforestry systems of *Theobroma cacao* and natural forests [35,36,37,38]. To this end, we established the following research questions: (1) Are the richness and composition of bryophyte communities influenced by microclimatic changes (i.e., canopy openness) in plantations and secondary forests? and (2) Can epiphytic bryophytes be used as indicators of *J. neotropica* plantations?

## 2. Materials and Methods

### 2.1. Study Area

**Figure 1:**
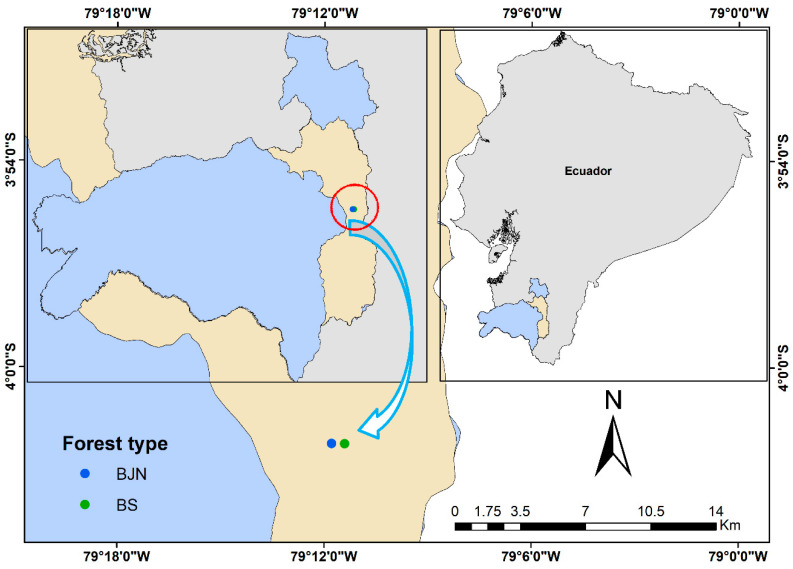
Study area in Loja Province, southern Ecuador, showing the location of the mixed *Juglans neotropica* plantation and secondary montane forests.

The research was carried out in two types of forest in the Universitary Park “Francisco Vivar Castro” (PUFVC), located south of the city of Loja, 5 km from the downtown area. The average annual temperature ranges between 15.6 and 16.6 °C; the average annual rainfall is 812.6 mm/year; the average relative humidity is 71.96% and the average evaporation is 111.33 mm. The two forest types occupy an approximate area of 22.41 ha. The mixed *J. neotropica* plantation is located in the lower part of the park, at an altitude of 2130 masl (Figure 1), with an area of 0.7 ha. This plantation is approximately 60 years old [45], where no thinning operations have been made, and is characterized by a very uniform structure, the predominant species being *J. neotropica*, mainly mixed with tree species of *Cedrela montana*, *Siparuna muricata*, *Inga fendleriana*, *Vibumum triphyllum*, *Streptosolen jamesonii*, *Palicourea heterochroma* and *Oreopanax rosei* [45]. The species is cultivated because it is an important timber species that produces high quality wood [46], and its nuts are edible and used as a colorant in the textile industry [47].

The montane forest is located at an altitude range of 2130–2520 masl, with an area of 99.13 ha and over 60 years of age [48]. The upper canopy is composed of *Alnus acuminata*, *Palicourea amethystina*, *Phenax laevigatus* and *Clethra revoluta* [48]. The mixed *J. neotropica* plantation and secondary forests are characterized by the absence of management activities. Fieldwork was carried out between October 2017 and March 2018.

### 2.2. Design and Data Collection

Five 20 m × 20 m plots were established in each of the two forest types (Table 1); in each plot, 10 trees with a diameter at breast height (DBH) > 10 cm were selected, for a total of 100 trees. In each tree, the presence and cover of bryophytes was recorded with 20 cm × 30 cm quadrats established at two heights from the base of the tree (50–100 cm; and 101–200 cm), and two orientations (north and south), for a total of 400 quadrats. The samples were identified in the Herbarium of the Universidad Técnica Particular de Loja (HUTPL), and the Universidad Nacional de Loja (LOJA) using general and specific keys [49,50,51,52]. Light conditions were recorded by measuring percent canopy openness, using five digital hemispherical photographs per plot. The distance between photographs within a plot was 5 m. Digital photographs were always taken on overcast days and at breast height (1.3 m), using a horizontally leveled digital camera (Nikon Coolpix 4500, Nikon, Madrid, España). The hemispheric photographs were analyzed with Gap Light Analyzer (GLA) version 2.0 [53].

### 2.3. Data Analysis

Species diversity in the two forest types was determined by evaluating the specific richness at tree level. Species richness was then analyzed using a generalized linear model (GLM) with a Poisson error distribution and a logarithmic link function [54]. Species composition was visualized using a non-metric multidimensional scaling analysis (NMDS), with the purpose of observing the similarity of bryophyte communities based on the Bray-Curtis distance and 999 Monte Carlo permutations. NMDS were conducted using the R package “vegan” [55]. Finally, to analyze the effect of environmental variables as forest type, light and DBH, a correlation between the two fitted axes and the environmental variables was performed with the “envfit” function. To determine which bryophyte species was associated with each forest type, we applied the indicator species analysis [56] using the IndVal function of the “labdsv” package [57]. The indicator value ranges from 0 (the species was absent from one forest type) to 1 (the species occurred in all trees of one forest type and was absent from other trees). All analyses were performed using R statistical software version 3.6.3 [58].

## 3. Results

### 3.1. Richness

**Figure 2:**
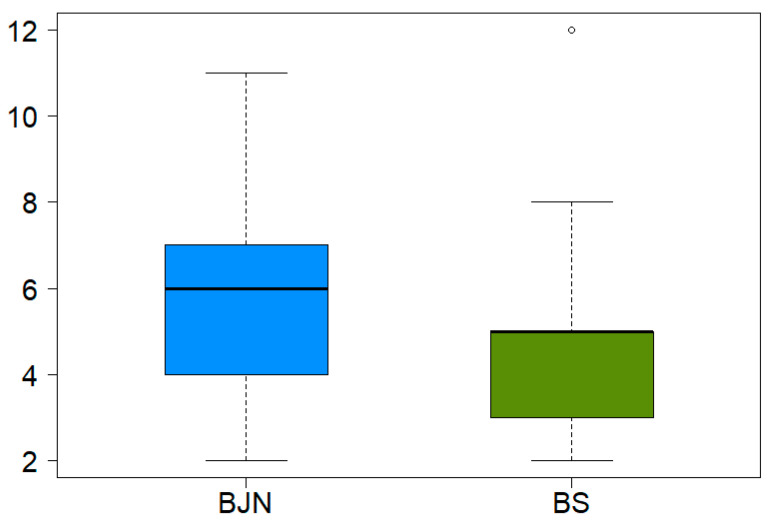
Box-plot representing bryophyte richness in the two types of forest: mixed *Juglans neotropica* plantation (BJN) and montane forests (BS); median richness (black horizontal bars).

A total of 55 species of epiphytic cryptogams (33 genera and 21 families) were recorded (Appendix A). The families with the highest number of species were Lejeuneaceae, Plagiochilaceae, Frullaniaceae and Meteoriaceae. At the forest level, the number of recorded species was similar in the two forest types, with 42 species (18 families and 23 genera) in the montane secondary forest and 41 species in the *J. neotropica* plantation (17 families and 22 genera). At tree level, the violin plot showed a higher number of species for the mixed *J. neotropica* plantation compared to secondary montane forests (Figure 2).

Forest type and DBH had significant positive effects on species richness. The mixed *J. neotropica* plantation showed the highest coefficients for bryophyte species richness, while the coefficients for secondary forest had the lowest values (Table 2). Conversely, canopy openness showed a significant negative effect on bryophyte richness (Table 2).

### 3.2. Species Composition

**Figure 3:**
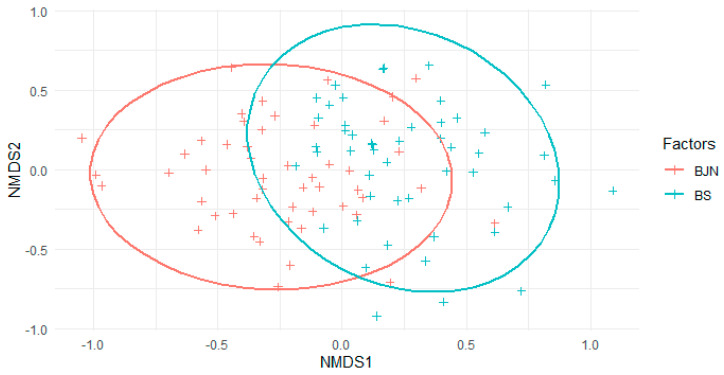
Nonmetric multidimensional scaling analysis ordination plot for the samples (trees) from the two types of forests (BS = Secondary forests; BJN = mixed plantations of *Juglands neotropica*).

The NMDS ordination showed that the community composition of epiphytic bryophytes is different in the two forest types (Figure 3).

The multivariate statistical analyses showed that epiphytic bryophyte composition was structured according to microclimatic changes, with a large component of variation (i.e., 34%) associated with canopy openness, followed by forest type and DBH, with 26% and 6%, respectively (Table 3).

### 3.3. Indicator Species

The analysis of indicator species determined four indicator species in the mixed platation of *J. neotropica*: *Radula tectiloba* with indicator value of 58.9, *Frullania ericoides* with 32, *Frullania riojaneirensis* with 30 and *Lejeunea deplanata* with 18, and four species in secondary forests: *Plagiochila raddiana* with indicator value of 55.5, *Porotrichum longirostre* with 39.4, *Pseudomarsupidium decipiens* with 15.5 and *Lophocolea bidentata* with 13.8.

## 4. Discussion

The results indicate that the richness and composition of epiphytic cryptogams were affected by forest type, mainly due to changes in canopy openness. Similarly, previous studies found an effect of canopy cover on the diversity of epiphytic bryophytes in montane forests [22,37,38,59,60]. We recorded a relatively high number of bryophytes in the mixed plantation of *J. neotropica* (41 species), which is similar to the 51 species reported for *Theobroma cacao* plantations [30], and higher than the 24 species found in monospecific forests of *Alnus acuminata* Kunth [26].

Species richness was influenced by forest type, with a higher number of species being recorded in the mixed *J. neotropica* plantation at the tree level compared with secondary forest. This is mainly due to the fact that the *J. neotropica* plantation presented more canopy openness compared to the secondary forest, which implies alterations in the microclimate with lower humidity and higher light availability. These changes favored the establishment of sun epiphytes of the genera *Frullania*, *Lejeunea* and *Radula*, which are better adapted to these environments, having functional characteristics adapted to conditions of excess light. Similar to our findings, several studies have documented that disturbed forests have a higher number of sun epiphytes, so the total richness is equal or even increases in disturbed forests when compared to natural forests [22,25]. On the other hand, the *J. neotropica* plantation appeared to be of minor conservation importance for the bryophytes with narrow microclimatic niches (drought-sensitive species: *Plagiochila raddiana* and *Porotrichum longirostre*). Similar results have been shown in cacao agroforests [35,36,37,38]. In addition, the studied *J. neotropica* plantation was established around 60 years ago, making it a mixed system with native species from secondary forests, which possibly explains why there is a similar number of species in the two forests. The composition of epiphytic bryophyte communities changes significantly in the secondary forest with respect to the mixed *J. neotropica* plantation, mainly related to more canopy openness, which is in accordance with several studies carried out in tropical areas [21,22,26,59]. Following the same pattern, the indicator species analysis (ISA) showed that *Radula tectiloba*, *Frullania ericoides* and *Frullania riojaneirensis* are good indicator species for *J. neotropica* plantations. Corroborating the findings, Acebey et al. [59] and Benitez et al. [26] point out that sun epiphytes are more dominant in secondary or disturbed forests, and they replace shade epiphytes. Thus, Gradstein [51] points out that these species are most common in open woodlands (i.e., drought tolerant epiphytic liverworts).

A different pattern can be seen in the secondary forest, which is characterized by more canopy cover, where the shade epiphytes (e.g., *Porotrichum* and *Plagiochila*) were dominant, due to the fact that these forests have higher humidity and less light availability. Thus, *Plagiochila raddiana* and *Porotrichum longirostre* were the best indicator species for secondary forests. Corroborating this pattern, several studies have documented that shade epiphytes are restricted to native and secondary forests that provide optimal microclimate conditions [21,22,25,26,60], because these species are sensitive to microclimatic changes due to their need for high humidity levels. In our case, the mixed plantation of *J. neotropica* presented a higher percentage of light (33.69%) that passes to the understory compared to the secondary forest (25.38%), which explains the absence of species with higher water needs. Similarly, Sporn et al. [37] and Ariyanti et al. [36] showed singnificant changes in bryophyte composition between cacao agroforests and natural forests, related to microclimatic changes (e.g., canopy cover). Finally, tree diameter had a significant effect on the richness and composition of bryophyte communities, related to increased substrate availability for species distribution and establishment. Similar to our results, Guerra et al. [60] and Gradstein and Culmsee [61] found that tree diameter is a key factor for the establishment and distribution of bryophytes in tropical forests, because large trees offer more surface area for the colonization of epiphytic bryophyte communities [26].

## 5. Conclusions

The diversity of bryophytes in mixed plantations of *J. neotropica* and secondary forests was conditioned by the canopy openness. Although the two habitat types showed a similar total richness at forest level, at tree level a higher richness of bryophytes (especially sun epiphytes) was recorded in the plantations compared with secondary forests. Although older mixed plantations (circa 60 years) of *J. neotropica* are colonized by native species and have a similar richness of bryophytes compared with secondary forests, these plantations do not harbour communities of species adapted to high humidity conditions in the same way secondary forests can. Since *J. neotropica* plantations do not provide a refuge for the local epiphytic bryophyte species with narrow microclimatic niches, natural forests are crucial to the conservation of the drought-sensitive forest bryophyte species.

## Figures and Tables

**Table 1:** Means of the environmental variables in the studied mixed *Juglans neotropica* plantation (BJN) and secondary montane forests (BS), with five plots of 20 m × 20 m (400 m^2^) within of each forest type in Ecuador. MTD = mean tree diameter.

Plot	Forests	Canopy Openess (%)	MTD (cm)
1	BJN	41.76	16.92
2	BJN	38.02	24.08
3	BJN	37.96	26.47
4	BJN	32.11	28.40
5	BJN	28.61	25.32
1	BS	21.86	17.37
2	BS	27.67	19.02
3	BS	26.56	24.17
4	BS	26.72	19.23
5	BS	24.09	14.55

**Table 2:** Results of the generalized linear models showing the effects of forest type (BJN—mixed *Juglans neotropica* plantation, BS—secondary montane forests), canopy openness and diameter at breast height (DBH) on the richness of epiphytic bryophytes. Coef.—coefficient, ES.—Stardar error *Z*.—*z* value, *P*—*p*-value.

	Coef.	ES	*Z*	*p*-Value
BJN	2.23537	0.39043	5.725	<0.0001
BS	−0.31825	0.12705	−2.505	0.01225
Canopy openness	−0.02284	0.01086	−2.104	0.03539
DBH	0.01085	0.00409	2.652	0.00801

**Table 3:** Squared correlation coefficients (*r*^2^) fitted on the first two axes of the NMDS ordination for host tree species, host tree traits and environmental factors. BJN—mixed *Juglans neotropica* plantation, BS—secondary montane forests, DBH—diameter at breast height.

	NMDS1	NMDS2	*r* ^2^	*p*-Value
Forests			0.261	0.000999
BJN	−0.2736	−0.0361		
BS	0.2792	0.0368		
Canopy openness	−0.94759	−0.3195	0.3426	0.000999
DBH	−0.5131	−0.85833	0.0643	0.042957

## Data Availability

Data is contained within the article.

## Appendix A

**Table A1:** Number of trees on which each species appears in two types of forests. (BS = Secondary forests; BJN = mixed plantations of *Juglans neotropica*). * denote threatened species in Ecuador [62].

Taxa	Forest of *Juglans neotropica* (BJN)	Secondary Forests (BS)
Liverworts		
Aneuraceae		
*Riccardia digitiloba* (Spruce) Pagan		1
Adelanthaceae		
*Pseudomarsupidium decipiens* (Hook.) Grolle	1	8
Frullanieaceae		
*Frullania brasiliensis* Raddi	6	1
*Frullania caulisequa* (Nees) Mont.	2	1
*Frullania ericoides* (Nees) Mont.	16	
*Frullania riojaneirensis* (Raddi) Ångstr.	15	
*Frullania subtilissima* (Nees ex Mont.) Lindenb.		1
Lejeuneaceae		
*Bryopteris filicina* (Sw.) Nees	1	
*Cheilolejeunea filiformis* (Sw.) W. Ye, R.L. Zhu & Gradst.		1
*Dicranolejeunea axillaris* (Nees & Mont.) Schiffn.	1	
*Drepanolejeunea cutervoensis* (Loitl.) Grolle		1
*Frullanoides densifolia* Raddi *subsp. densifolia.*	1	
*Lejeunea cerina* (Lehm. & Lindenb.) Lehm. & Lindenb.	9	15
*Lejeunea deplanata* Nees	9	
*Lejeunea flava* (Sw.) Nees	1	1
*Lejeunea laetevirens* Nees & Mont.	17	12
*Lejeunea ramulosa* Spruce	3	
*Marchesinia brachiata* (Sw.) Schiffner	1	
*Microlejeunea acutifolia* Steph.	1	
*Microlejeunea bullata* (Taylor) Steph.		1
Lophocoleaceae		
* *Leptoscyphus autoicus* (J.J. Engel & Gradst.) Vanderp. and Gradst.		1
*Lophocolea bidentata* (L.) Dumort.	1	7
*Lophocolea muricata* (Lehm.) Nees		4
Metzgeriaceae		
*Metzgeria dorsipara* (Herzog) Kuwah.	2	1
*Metzgeria leptoneura* Spruce	1	3
Plagiochilaceae		
*Plagiochila aerea* Taylor		1
*Plagiochila bifaria* (Sw.) Lindenb.		1
*Plagiochila cristata* (Sw.) Lindenb.	9	4
*Plagiochila diversifolia* Lindenb. & Gottsche	6	3
*Plagiochila gymnocalycina* (Lehm. & Lindenb.) Mont. and Nees	2	1
*Plagiochila raddiana* Lindenb.	29	36
Porellaceae		
*Porella brachiata* (Taylor) Spruce	2	2
*Porella crispata* (Hook.) Trevis.	3	3
Radulaceae		
*Radula episcia* Spruce	3	7
*Radula gottscheana* Taylor	7	8
*Radula tectiloba* Steph.	34	7
Mosses		
Bryaceae		
*Bryum apiculatum* Schwägr		1
Calymperaceae		
*Syrrhopodon incompletus* Schwägr.	7	5
Cryphaeaceae		
*Cryphaea jamesonii* Taylor	3	
Daltoniaceae		
*Adelothecium bogotense* (Hampe) Mitt.		1
Dicranaceae		
*Campylopus flexuosus* (Hedw.) Brid.	1	
Fabroniaceae		
*Fabronia ciliaris* (Brid.) Brid.	1	
Meteoriaceae		
*Meteoridium remotifolium* (Müll. Hal.) Manuel	6	5
*Squamidium leucotrichum* (Taylor) Broth.	3	5
*Squamidium nigricans* (Hook.) Broth.	2	3
*Zelometeorium recurvifolium* (Hornsch.) Manuel		13
Mniaceae		
*Plagiomnium rhynchophorum* (Hook.) T.J. Kop.		1
Neckeraceae		
*Neckeropsis undulata* (Hedw.) Reichardt	12	11
*Porotrichum filiferum* Mitt.		4
*Porotrichum longirostre* (Hook.) Mitt.	15	26
Orthotrichaceae		
*Macromitrium richardii* Schwägr.	2	
Sematophyllaceae		
*Acroporium pungens* (Hedw.) Broth.	1	2
*Sematophyllum subsimplex* (Hedw.) Mitt.	14	7
Thuidiaceae		
*Thuidium peruvianum* Mitt.	35	25
*Thuidium tomentosum* Schimp.	2	2
